# The diagnostic role of the β-hCG discriminatory zone combined with the endometrial pattern for ectopic pregnancy in Chinese women

**DOI:** 10.1038/s41598-019-50151-x

**Published:** 2019-09-24

**Authors:** Qi Lu, Yiwei Wang, Xiao Sun, Yuhong Li, Jing Wang, Yun Zhou, Yudong Wang

**Affiliations:** 1 Department of Gynecology, Jinshan Hospital of Fudan University, 1508 Longhang Rd., Shanghai, 201508 China; 2 Department of Gynecology, International Peace Maternity and Child Health Hospital, Shanghai Jiaotong University School of Medicine, 910 Hengshan Rd., Shanghai, 200030 China; 3 Department of Ultrasound in Obstetrics and Gynecology, International Peace Maternity and Child Health Hospital, Shanghai Jiaotong University School of Medicine, 910 Hengshan Rd., Shanghai, 200030 China

**Keywords:** Diagnostic markers, Urogenital reproductive disorders

## Abstract

Previous studies have regarded the discriminatory serum β-hCG zone (DSZ) as a valuable tool for the diagnosis of ectopic pregnancy (EP). However, the wide range of the DSZ makes achieving a clinical diagnosis of EP difficult, and these reports do not indicate whether the DSZ is suitable for an EP diagnosis in Chinese women. Several studies have indicated that the endometrial pattern in patients with EPs is different from that in patients with intrauterine pregnancies (IUPs). The aims of this study were to define the DSZ cutoff value for Chinese women, test whether the endometrial pattern is a suitable predictor for EP, and assess the diagnostic value of these indicators. We enrolled participants with IUPs or EPs with abdominal pain and/or vaginal bleeding, and serum β-hCG level measurements and transvaginal ultrasound (TVS) were performed to assess the diagnostic value of the indicators for EP. The sensitivity and specificity for identifying an EP were improved by combining the DSZ, endometrial thickness and trilaminar pattern indexes. The results of this study might be helpful toward providing further options for the diagnosis of EP, especially for patients without hemoperitoneum or colporrhagia.

## Introduction

Ectopic pregnancy (EP) is a common and potentially lethal emergency in the first trimester of pregnancy^1^. The overall prevalence of EP is nearly 1% for all pregnant women^2^, and a higher incidence is associated with pelvic inflammatory disease, sexually transmitted diseases and the utilization of assisted reproductive technology^3^. The maternal mortality rate for Chinese women with EP has been reported to be approximately 16/1 million women^4^. Although great advances in the diagnostic and therapeutic management of EP have been made over the last two decades, misdiagnosis and delayed intervention remain the leading clinical causes of maternal death due to a lack of rapid and accurate diagnostic methods^5^.

The guidelines of the Royal College of Obstetricians and Gynecologists (RCOG) in 2016 indicate that transvaginal ultrasound is the most important diagnostic tool of choice for tubal ectopic pregnancy and that laparoscopy is no longer the gold standard^6^. Our previous study showed that the ratio of the β-hCG level in the blood at the location of the pregnancy (hemoperitoneum or vaginal blood) to the β-hCG level in venous serum can be used as a diagnostic indicator of EP^7,8^. However, this method is not suitable for patients without hemoperitoneum or vaginal bleeding. Hence, new diagnostic methods need to be developed for the EP diagnosis. Recently, several diagnostic tests have been used to evaluate patients with suspected EP, including β-hCG measurements and transvaginal ultrasound (TVS).

TVS may reveal an intrauterine pregnancy (IUP), an EP, a molar pregnancy, or none of the above (also known as “pregnancy of unknown location”), which occurs in 10% to 30% of symptomatic patients^9,10^. An extrauterine gestational sac containing a yolk sac and/or embryonic pole can be identified by TVS, and an EP can be diagnosed according to the RCOG guidelines^6^. However, the abovementioned findings on TVS can only be observed in a few clinical cases. In most cases, an empty uterine cavity, an extrauterine mass and anechoic fluid in the pouch of Douglas are visualized on TVS, which can help to indicate a diagnosis of EP. Sometimes the sac is too small to be visualized during the initial ultrasound examination in the early process of an EP. Recently, several studies have shown that the endometrial thickness associated with EP is much lower than that associated with IUP and that EP might result in intracavitary fluid as well as trilaminar, homogeneous and heterogeneous patterns^10,11^. To improve the accuracy of the EP diagnosis, more characteristics of TVS should be taken into account, including the thinner endometrium and the trilaminar pattern.

Moreover, the RCOG guidelines emphasize that the β-hCG level is useful for planning the management of ultrasound-visualized EP^6^. Kadar *et al*. introduced the concept of a discriminatory serum β-hCG zone (DSZ)^12^. According to the DSZ, an EP might be indicated when the concentration of serum β-hCG is beyond the threshold^13^. However, in the clinical setting, the situation is far more complicated. The DSZs in the previous literature are not consistent between studies, in which the DSZ ranges from 1000 to 3500 IU/L^14–17^. The wide range increases the difficulty in achieving a clinical diagnosis, and it is unclear whether the DMZ is suitable for Chinese women.

The purpose of this study was to determine the DSZ for Chinese people and assess the diagnostic value of the DSZ, endometrial thickness and trilaminar patterns for EP.

## Results

### Clinical characteristics of the two groups

**Figure 1:**
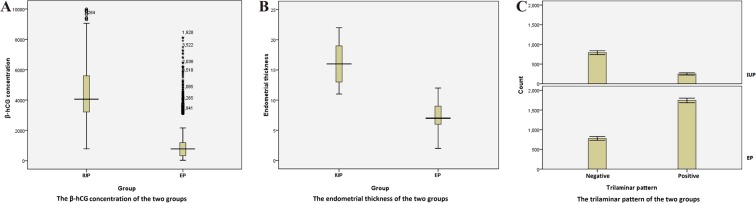
The graphical representation of the 3 parameters of the two groups is shown. The boxplot graph is used to demonstrate the distributions of continuous variables according to the serum β-hCG concentration (**A**) and the endometrial thickness (**B**), and the minimum values, first quartiles, medians, third quartiles and maximum values are presented. The bar chart is used to represent the categorical variables in the two groups, namely, the number of patients with a trilaminar pattern (**C**).

In total, the study sample consisted of 3558 participants with IUPs (n = 1038) or EPs (n = 2520). The demographic data and clinical characteristics of the two groups are summarized in Table 1 and Fig. 1A–C. The differences in age and menstrual cycle duration between the EP and IUP groups were not statistically significant (*P* > 0.05). The β-hCG concentration, abdominal pain, vaginal bleeding, endometrial thickness and trilaminar pattern were significantly different between the two groups (*P* < 0.001).

**Table 1:** Characteristics of the patients in the ectopic pregnancy group and the intrauterine pregnancy group.

	EP group (n = 2520)	IUP group (n = 1038)	*P* value
Age (years)	31 (23~40)	30 (23~39)	NS
Menstrual cycle duration (days)	40 (35~47)	41 (33~47)	NS
β-hCG concentration (IU/L)	1052 (470~3933)	4328 (1326~8526)	<0.001
Vaginal bleeding (yes/no)	1753/767	228/810	<0.001
Abdominal pain (yes/no)	1423/1097	373/665	<0.001
**Endometrial pattern**			
Endometrial thickness (mm)	7 (4~19)	16 (4~20)	<0.001
Trilaminar pattern (yes/no)	1742/778	248/790	<0.001

Data are presented as the number (%) or as the median [5%, 95%]. EP represents ectopic pregnancy. IUP represents intrauterine pregnancy. NS represents *P* > 0.05.

### Single index for diagnosis

**Figure 2:**
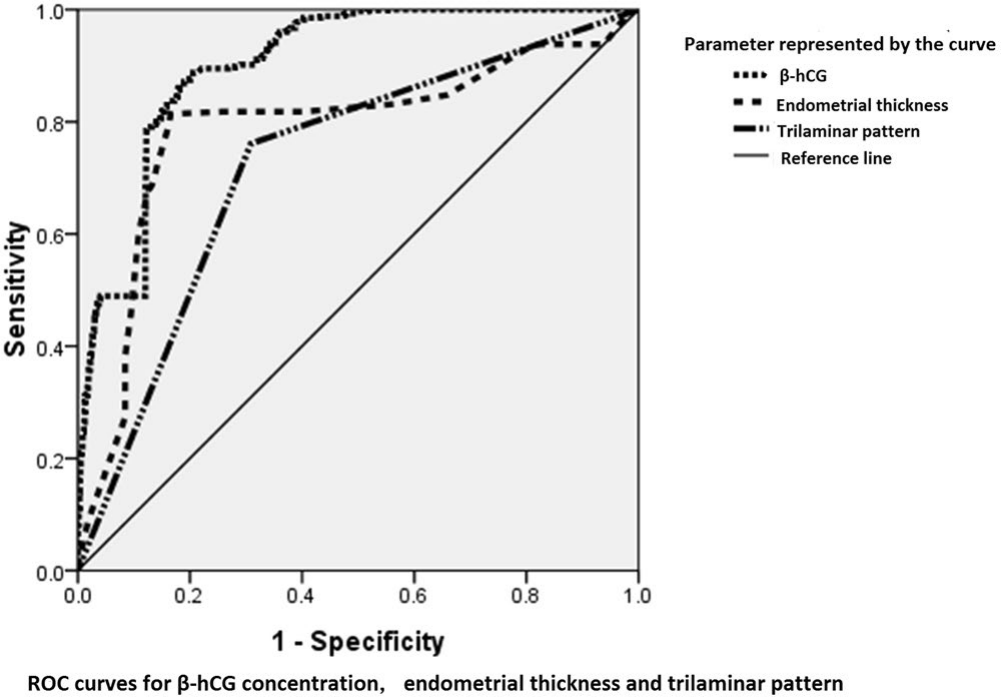
The diagnostic value of a single index for EP was evaluated by using a ROC curve, which was constructed to measure sensitivity, specificity, and the area under the curve with the 95% CI. The results of the ROC analysis of each index, namely, the discriminatory serum β-hCG zone (DSZ), endometrial thickness and trilaminar pattern, are presented.

We evaluated the diagnostic value of a single index including the DSZ, endometrial thickness and trilaminar pattern for EP by performing a receiver operating characteristics (ROC) curve analysis. The results of the ROC analysis are presented in Table 2 and Fig. 2.

**Table 2:** The ROC analysis of a single index for the diagnosis of EP.

	AUC	95% CI	*P* value	sensitivity	specificity
DSZ	0.901	0.891~0.911	<0.001	0.875	0.807
Endometrial thickness	0.790	0.772~0.809	0.009	0.814	0.835
Trilaminar pattern	0.726	0.708~0.744	<0.001	0.691	0.761

ROC represents receiver operating characteristic. AUC represents the respective area under the curve. DSZ represents the discriminatory serum β-hCG zone.

The ROC curves showed that the cutoff value for β-hCG in EP was 1511 IU/L (AUC: 0.901, 95% CI: 0.891–0.911, sensitivity: 87.5%, and specificity: 80.7%). Thus, we chose 1500 IU/L as the DSZ for EP in this study. The cutoff value for endometrial thickness was 10.5 mm (AUC: 0.790, 95% CI: 0.772–0.809, sensitivity: 81.4% and specificity: 83.5%). We chose 10 mm as the endometrial thickness threshold for EP. The sensitivity and specificity of the trilaminar pattern for the EP diagnosis were 69.1% and 76.1%, respectively (AUC: 0.726, 95% CI: 0.708–0.744).

### Combined indexes for diagnosis

**Figure 3:**
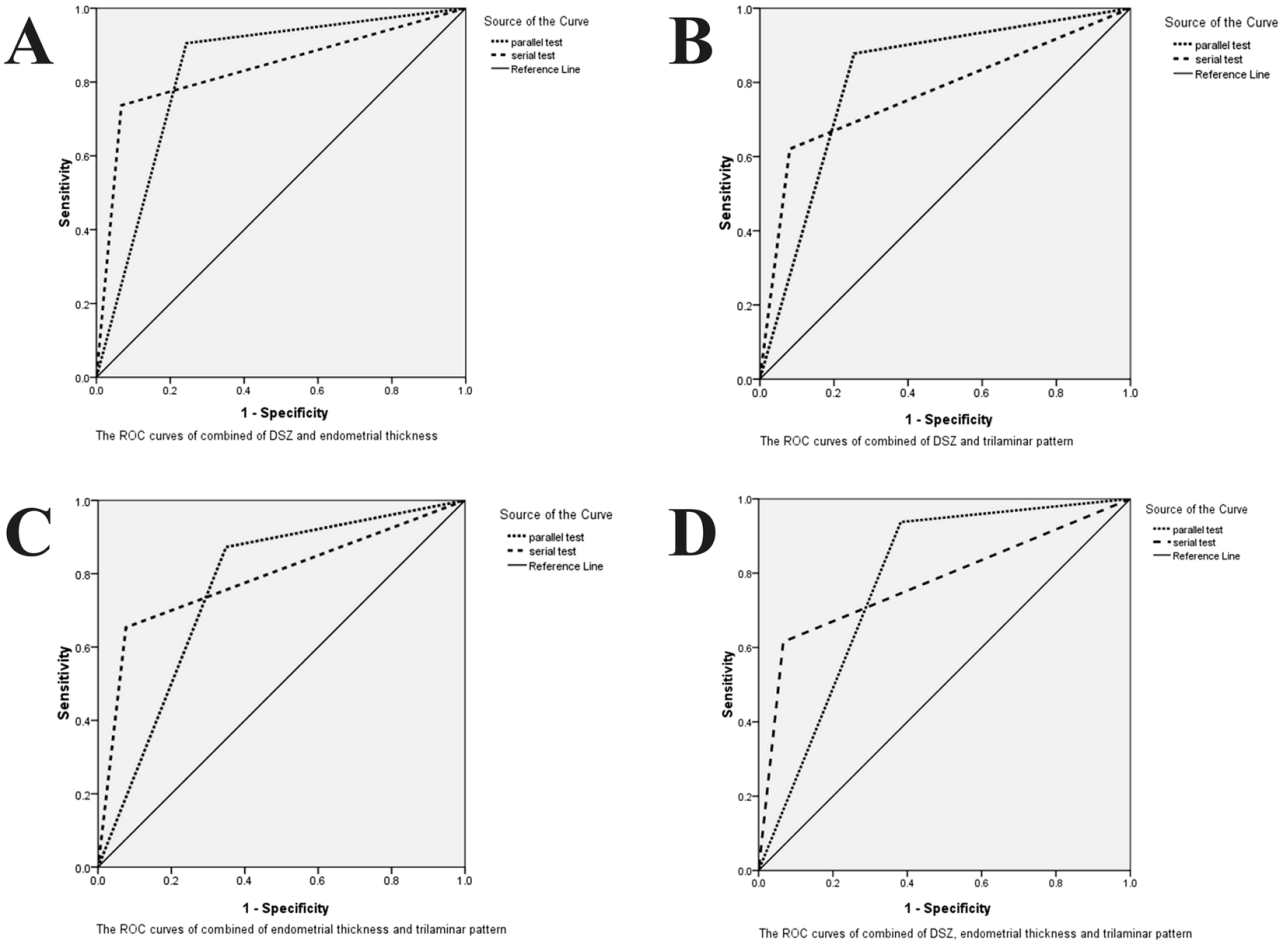
The combination of a single index with another was performed using the parallel and serial tests to improve the accuracy of EP diagnosis. The parallel test result is considered positive when only one index is positive and negative when all indexes are negative. The serial test result is considered positive only when all test results are positive and negative when one result is negative. The ROC analysis of the combined indexes, namely, the DSZ and endometrial thickness (**A**), the DSZ and trilaminar pattern (**B**), the endometrial thickness and trilaminar pattern (**C**), and all three indexes (**D**), is presented.

To improve the accuracy of diagnosis, we generated a single index to identify EP and IUP by using the parallel and serial test. The parallel test result was regarding as positive when only one index was positive and negative when all indexes were negative. The serial test result was regarded as positive only when all test results were positive and negative when one result was negative. The parallel test improved the sensitivity of detection, and the serial test improved the specificity of detection compared with the single index. The results of the ROC analysis of the combined indexes are shown in Table 3 and Fig. 3A–D.

**Table 3:** The ROC analysis of the combined indexes for the diagnosis of EP.

	AUC	95% CI	*P* value	sensitivity	specificity
**DSZ and Endometrial thickness**					
parallel test	0.831	0.814~0.847	<0.001	0.905	0.756
serial test	0.835	0.821~0.849	<0.001	0.737	0.933
**DSZ and Trilaminar pattern**					
parallel test	0.815	0.795~0.829	<0.001	0.878	0.746
serial test	0.770	0.754~0.786	<0.001	0.621	0.919
**Endometrial thickness and Trilaminar pattern**					
parallel test	0.762	0.881~0.918	<0.001	0.873	0.651
serial test	0.789	0.74~0.781	<0.001	0.654	0.924
**DSZ**, **Endometrial thickness and Trilaminar pattern**					
parallel test	0.778	0.758~797	<0.001	0.938	0.618
serial test	0.775	0.759~0.790	<0.001	0.615	0.934

ROC represents receiver operating characteristic. AUC represents the respective area under the curve. DSZ represents the discriminatory serum β-hCG zone.

The sensitivity of the parallel test (criteria: a β-hCG value above 1500 IU/L or endometrial thickness above 10 mm as the diagnostic threshold) was 90.5%. The specificity of the serial test (criteria: a β-hCG value above 1500 IU/L and endometrial thickness above 10 mm as the diagnostic threshold) was 93.3% (Table 3, Fig. 3A). With the combination of β-hCG value and trilaminar pattern, the sensitivity of the parallel test and the specificity of the serial test were 87.8% and 91.9%, respectively (Table 3, Fig. 3B). The sensitivity of the parallel test and the specificity of the serial test with the combination of the endometrial thickness and trilaminar pattern were 87.3% and 92.4%, respectively (Table 3, Fig. 3C). Moreover, with the combination of the three indexes, the sensitivity of the parallel test and the specificity of the serial test were 93.8% and 93.4%, respectively (Table 3, Fig. 3D).

## Discussion

EP is one of the leading causes of maternal mortality in early pregnancy. The mortality rate of EP is estimated to be 16.9 per 1,000,000 EPs in the Chinese population^4^, and EP is responsible for 4 to 10% of pregnancy-related deaths around the world^2^. The accurate and expeditious diagnosis of EP is of particular concern.

The identification of serum biomarkers is an important goal in the diagnosis of EP, especially for the detection of early stage EP. Serum β-hCG is the only biomarker that is used routinely and widely in clinical practice. β-hCG alone is not sensitive enough for an EP diagnosis, but β-hCG is helpful toward identifying patients who require closer surveillance for early pregnancy failure. The expected hCG rise over 48 hours for a viable IUP is at least 53%^18^. However, for EPs, this method often requires repeated follow-up over several days, during which there is a prolonged risk of tubal rupture. Other biomarkers, such as progesterone, PAPP-A, SP-1, Inhibin A, VEGF, Activin A and ADAM-12, are unable to differentiate EP from abnormal IUP with sufficient accuracy, and these markers need to be studied further before they can be used in clinical practice^19^.

Our previous study showed that a hemoperitoneum-to-venous-serum β-hCG ratio that is greater than 1.0 can be used as an indicator for an immediate diagnosis of EP with hemoperitoneum. Furthermore, we demonstrated that a vaginal-blood-to-venous-serum β-hCG ratio that is greater than 1.0 can be used as an indicator for EP with vaginal bleeding^7,8^. However, this method is not suitable for patients without hemoperitoneum or vaginal bleeding.

TVS has become an important tool in the diagnosis of EP^20^. EP is being diagnosed with greater accuracy by TVS, which is a rapid, simple and noninvasive method. Initially, the DSZ for the possible occurrence of an EP was defined as a β-hCG level greater than 6500 IU/L when a gestational sac was not detected by transabdominal ultrasound^12^. With the advances in TVS, the DSZ was redefined, resulting in the current range of 1000 IU/L to 3500 IU/L^14–17^. TVS is sensitive and specific for an EP diagnosis when the β-hCG level is above the DSZ with positive identification of an adnexal mass. However, the EP diagnosis by TVS is much more complicated in the clinical setting. The diagnosis is less evident when the β-hCG level is below the DSZ and when the adnexal ultrasonographic findings are inconclusive. A more accurate and safe diagnostic method is still required for EP.

In this study, 3558 Chinese women with IUPs (n = 1038) or EPs (n = 2520) were enrolled for the assessment of the diagnostic accuracy of the DSZ, endometrial thickness and the trilaminar pattern for EP. Our study showed that a DSZ value of 1500 IU/L and an endometrial thickness of 10 mm were acceptable as the threshold for the diagnosis of EP in Chinese women.

In contrast to that in previous studies, the DSZ threshold in our study was included in the reported range. The sensitivity of the DSZ in our previous study was 87.5%; this value was 77% in the study by Wang, *et al*. and 87% in the study by Shaunik, *et al*.^21,22^. The specificity was 80.7% in our study and 99% in the study by Murray^23^. A previous study showed that for a woman whose DSZ ranged from 2000 to 3000 IU/L, the likelihood of a viable IUP was approximately 0.5%^24^. A false-positive result might do harm to normal pregnancies. Based on the cutoff value of 1500 IU/L in the Chinese population in our study, the likelihood of a false-positive result was not obviously decreased. Furthermore, as an indirect test, a single β-hCG measurement is unreliable for an independent diagnosis of EP. Therefore, we also sought other indexes to improve the diagnostic accuracy of EP.

Several studies have indicated that the endometrial thickness of EP (5.95 ± 0.35 mm) is much lower than that of IUP (13.42 ± 0.68 mm), while 8 mm is the cutoff endometrial thickness value for the EP diagnosis. It has been hypothesized that EP is significantly correlated with a thin endometrium and that an endometrial strip ≤8 mm might result in either EP or spontaneous abortion^25,26^. A thickened endometrium is less common in EP because of the poor development of the villus tissue, adverse environment for embryo implantation and low level of β-hCG excretion. Our study showed that the sensitivity and specificity of the endometrial thickness assessment were 81.4% and 83.5%, respectively, when the endometrial thickness was 10.5 mm. One group documented a similar observation that for every 1-mm increase in endometrial thickness, there is a 27% decrease in the chances of an EP, which might explain the higher sensitivity and specificity in our study^26^. However, the sole evaluation of endometrial thickness is still inadequate in the diagnosis of EP.

EP results in intracavitary fluid as well as trilaminar, homogeneous and heterogeneous patterns^27^. During the EP process, the trilaminar pattern always changes under ultrasonic inspection^28,29^. Hammoud *et al*. retrospectively investigated 403 patients and reported that the trilaminar pattern had a 94% specificity and 38% sensitivity for the prediction of an EP^11^. Our study indicated that the AUC of the trilaminar pattern was 0.726 with a sensitivity of 69.1% and a specificity of 76.1%. The trilaminar pattern seems to present a certain clinical value for the early diagnosis of EP.

To improve the accuracy of diagnosis, we combined three parameters into a single index (DSZ, endometrial thickness and trilaminar pattern) to diagnose EP and IUP. The sensitivity and specificity were improved when we combined any two of the three indexes. Furthermore, when the three indexes were combined, the sensitivity of the parallel test and the specificity of the serial test were 93.8% and 93.4%, respectively, which were better than those of a single index and combination of any two indexes. Our previous work demonstrated that for patients with EPs with peritoneal effusion or colporrhagia, the pregnancy-location-blood-to-venous-blood β-hCG ratio could assist in the diagnosis, whereas for EP patients with negative hemoperitoneum or colporrhagia, the DSZ and endometrial pattern would be more suitable for diagnosis.

In conclusion, this study is the first to determine the DSZ (1500 IU/L) for EP in Chinese women and to combine the DSZ with the endometrial pattern to provide further options for the EP diagnosis. The conclusion of this study might be helpful toward providing a more accurate and improved diagnostic process for EP, especially for patients who are negative for hemoperitoneum or colporrhagia. The sample size of our study is larger than that of previous studies. However, there are also several limitations in this study. The main limitation was its retrospective study design. Another limitation was the exclusion of patients with unstable vital signs from the study, which might have caused selection bias. It is necessary to carry out further studies with a prospective design and a larger sample size to confirm our conclusion, which might be helpful toward improving the diagnostic accuracy of EP, avoiding unnecessary surgery for IUP patients who desire fertility preservation, and preventing the adverse outcomes of EP, such as fatal or massive hemorrhage.

## Methods

### Clinical sample

The data were retrieved from the database of International Peace Maternity and Child Health Hospital, Shanghai Jiaotong University, China, covering the period from January 2013 to January 2016. The hospital is one of the largest obstetric care centers in Shanghai, with approximately 16,000 annual deliveries. The inclusion criteria of all subjects were as follows: women of childbearing age, abdominal pain or vaginal bleeding, a history of amenorrhea, and a positive pregnancy test (urine β-hCG). Patients were excluded if they met following exclusion criteria: gynecological tumors or other serious diseases, serious complications, unstable vital signs, and serum β-hCG measurements and TVS data that were not obtained on the same day.

The definitive diagnosis was classified as EP or IUP. The gold standard for EP was based on a pathological diagnosis when surgical intervention or diagnostic curettage was performed. In the absence of surgical intervention, the gold standard was based on signs on transvaginal ultrasound indicating an adnexal mass that was separate from the ovary, that comprised a gestational sac containing a yolk sac or that comprised a gestational sac with a fetal pole (with or without a fetal heartbeat)^6^. The gold standard for IUP was based on signs on transvaginal ultrasound indicating a gestational sac containing a yolk sac or a gestational sac with a fetal pole (with or without a fetal heartbeat) in the uterine cavity.

When the diagnosis remained obscure after a clinical evaluation and pelvic ultrasonography, patients were managed with a follow-up regimen that consisted of repeated measurements of serum β-hCG levels and ultrasonography every 48 hours. Follow-up consultations were provided and continued according to the patient’s condition until a final diagnosis and treatment were achieved.

This study was approved by the Regional Ethics Committee of International Peace Maternity and Child Health Hospital, Shanghai Jiaotong University, China. All methods were performed in accordance with the relevant guidelines and regulations, and informed consent was obtained from all participants.

### Measurements

The general information of the patient was gathered, and a thorough examination was conducted, including serum β-hCG measurements and TVS scanning. The serum β-hCG concentration was measured with Bayer’s ADVIA Centaur automated chemiluminescence immunoassay system and the test kits for total serum β-hCG in the laboratory of International Peace Maternity and Child Health Hospital. The measurements of endometrial thickness and assessments of the endometrial pattern were performed by transvaginal ultrasonography with Doppler ultrasound after the patients had completely emptied their bladders. Endometrial thickness was measured in a median longitudinal plane of the uterus as the maximum distance between the endometrial-myometrial interface of the anterior to posterior wall of the uterus. The trilaminar pattern was defined as the outer line of a high echo formed between the endometrium and the medial layer of the uterus, and the centerline of a high echo was clearly visible from the surfaces of two layers of the endometrium. All sonograms, including images and video clips, were retrospectively reviewed by a single certified sonographer for the diagnosis of the presence and characteristics of an EP.

### Statistical analysis

Data collection and analyses were undertaken by independent researchers. Data are expressed as the mean ± SD values or as the median and range according to the distribution. The chi-square test was used to compare differences between the categorical variables (vaginal bleeding, abdominal pain and the trilaminar pattern). The normal distribution test was performed for continuous variables, including age, menstrual cycle duration, serum β-hCG concentration and endometrial thickness. If the continuous variables were not in accordance with a normal distribution, then the nonparametric test was used to analyze differences between two groups. The boxplot graph was used to measure the distribution of continuous variables, which presented the minimum value, the first quartile, the median, the third quartile and the maximum value. The bar chart was used to compare categorical variables. A ROC curve was constructed to measure sensitivity, specificity, and the respective areas under the curve (AUCs) with the 95% CIs. Parallel and serial tests were used to evaluate the diagnostic value of the combined indexes. The significance level for all analyses was *P* < 0.05. Statistical analysis was performed using the Statistical Package for Social Sciences (SPSS Inc., Chicago, IL, USA).
